# On Strictures of the Urethra

**Published:** 1852-12

**Authors:** G. J. Guthrie


From the London Lancet of June.

*On Strictures of the Urethra, by* G. J. Guthrie, Esq.

1. A hard and elastic, or intractable stricture is never permanently cured by dilatation, or by the application of caustic, although it may be materially relieved by the regular periodical use of a dilating instrument.

2. That the division of an old, hardened, or elastic stricture through the perineum is not usually followed by a permanent cure, although it is always attended by immediate relief. The disease being apt to return unless a solid sound or a catheter is occasionally passed to prevent it.

3. That the operation of dividing the perineum and urethra in such cases is sometimes attended by severe hæmorrhages, by fever, and is occasionally followed by fistulous openings, giving rise to much inconvenience.

4. That such division does, in some instances, effect a permanent cure.

5. That the division of the urethra through the external parts should never be attempted in any portion of it anterior to the bulb, such operation not being necessary; for the narrowest stricture of the pendulous or movable part may always be divided internally with much less comparative danger than by the external incision, inasmuch as the instrument can be guided through this part by the finger and thumb of the left hand of the surgeon with a certainty almost unerring.

6. That the stricture considered by all surgeons as the most important and difficult of cure—viz., at the termination of the bulbous portion of the urethra—may always be divided, when impassable, by a *straight* instrument, and, in general, more easily, than by a *curved* one; the use of which is founded on the erroneous belief that the stricture is situated in the membranous part of the urethra, instead of being, as it is anterior to it.

7. That the division of a stricture should, if possible, be effected by an instrument passed through it, and cutting from behind forwards, rather than from before backwards, although a combination of both methods will frequently be necessary to insure success.

8. The division of a stricture by these means will not always insure a permanent cure if more than the mucous membrane is implicated, unless such parts be divided also.

9. That in cases of intractable stricture, the mucous membrane, the inner layer of involuntary muscle, and the elastic tissue external to it, should be divided, when the operation is done from within, but not the outer layer of muscular fibres, which should remain as a barrier between the stream of urine and the common integuments of the external parts—an accuracy of division not always to be attained: whence, perhaps, the difficulty of effecting a permanent cure.

10. That when a permanent cure is effected in these cases, the divided elastic wall of the urethra is not re-united by a structure exactly similar to itself, but by common areolar tissue, rendering the part more dilatable under the pressure of the stream of urine; the formation of which dilatation can be aided during the progress of the cure by pressing on the divided part with the point of a solid instrument passed daily for the purpose of preventing, if possible, that contraction which always takes place during the process of cicatrization; a proceeding which cannot be advantageously adopted when the parts are divided through the perineum, lest it should encourage the formation of a fistulous opening, to which there is always a tendency.

11. That in cases of intractable stricture accompanied by one or more fistulous openings in the perineum, in *young persons*, or of middle age, the operation through the external parts, or along the urethra, may be resorted to at the pleasure of the surgeon with an equal chance of success, provided the division of the obstruction or bank preventing the free passage of the urine be effectually divided, the *sine qua non* of the operation.

12. That the operation within the urethra, should always be preferred in *elderly* persons, particularly if somewhat stout or fat, as less likely to create severe constitutional disturbance; and if this operation should fail from any cause, it by no means interferes with the due performance of the other through the perineum, which in serious cases then becomes imperative, as the last resource capable of giving relief.

